# Managerial Perception of Risk in an Organization in a Post-COVID-19 Work Environment

**DOI:** 10.3390/ijerph192214978

**Published:** 2022-11-14

**Authors:** Tomasz Ewertowski, Marcin Butlewski

**Affiliations:** Faculty of Engineering Management, Poznan University of Technology, Rychlewskiego Str., 60-965 Poznan, Poland

**Keywords:** risk perception, risk management, occupational health, COVID-19, crisis management, business continuity management, organizational resilience, operational risk

## Abstract

The COVID-19 pandemic drew the attention of all industries and organizations to the importance of comprehensive preparation for various types of crises and disruptions. Without proper risk management for crisis situations, it is impossible to talk about organizational resilience, maintaining organizational continuity, or ensuring the company’s ability to protect workers’ lives and health in a crisis. While the COVID-19 pandemic is rapidly reshaping the work environment, significant challenges related to risk management are emerging. The purpose of this research paper is to examine the impact of a pandemic on the risk perception in an organization by managers of all three levels (strategic, operational, and line level) and to examine the impact of broadly understood risk management on organizational performance. For the examination of operational risk perception, empirical research was conducted in Polish enterprises. The methodology of the survey is based on a questionnaire of operational risk and risk management perception in a post-COVID-19 work environment. According to the survey results, risk management was generally perceived better than the level of operational risk, compared to the period before the pandemic. Therefore, a substantial improvement in risk management during the crisis allowed the surveyed organizations to cope with the pandemic, and even slightly enhance their performance. Organizations have been able to achieve their goals mainly by slightly reducing risk appetite and lowering the tolerable risk level threshold. Even so, organizations have improved their ability to adapt and seize opportunities.

## 1. Introduction

Despite the statement of the head of the World Health Organization (WHO), who claimed at the end of September 2022 that the world has never been in a better position to end the COVID-19 pandemic, it is evident that the COVID-19 pandemic has caused a crisis in many dimensions. The epidemic has caused the death of over 6.5 million people, which is 0.08% of the world’s population [1]. This puts this pandemic in a distant place in the array of diseases affecting the human race [2]. Despite this, the pandemic and the way it was dealt with have had a big influence not only on healthcare systems, but also on global economies. Of course, it cannot be stated that the pandemic has already finished. However, one can already talk about the post-COVID-19 era due to our knowledge, statistics, and the optimistic assessment of the head of the WHO. One of the biggest winners of the new situation is, of course, risk management. The pandemic has drawn the attention of all industries and organizations to the importance of comprehensive preparation for various types of crises and disruptions. Without a proper approach towards risk management in a crisis, it is impossible to talk about organizational resilience, maintaining organizational continuity, and ensuring a company’s ability to protect workers’ lives and health in a crisis. Moreover, in order to manage the constantly increasing dynamics and hostility of the environment, companies need to transform. This transformation is required to maintain the sustainability of their products, processes, organization, and strategies [3]. In an extremely competitive market, only the fittest will survive. The coronavirus pandemic has interrupted labor markets and has had a big influence on workplace dynamics, with changes and new arrangements creating the so-called “new normal” work environment [4]. This is characterized, among other factors, by flexible work arrangements. Although hybrid and remote working are more popular in the post-pandemic period for non-manual work, traditional work practices remain. The big challenges are work–life balance (WLB), health and well-being [5], and thus the relationship of individuals to work. The coronavirus disease pandemic has demanded adjustments and changes from all levels of managerial staff. With fewer opportunities for in-person interactions in the workplace, managers need to focus on establishing and developing relationships with their subordinates to support and enhance the WLB of employees [6,7]. This new work environment has become even more complex than before, and uncertain in the context of new challenges caused by broken supply chains, increased insecurity and, as a consequence of many other factors, an unstable economy. The new work challenges thus characterized can be called a post-COVID-19 work environment. Therefore, this has to be managed by a risk assessment approach.

The purpose of this study is to examine the impact of a pandemic on the organizational risk (including occupational risk) perception in an organization by managers of all three levels (strategic, operational, and line) and to examine the impact of broadly understood risk management on organizational performance in a post-COVID-19 work environment. Managers were chosen as research subjects because they are recognized as risk experts who have sufficient experience and knowledge about risk assessment processes. On the other hand, non-expert workers tend to view risks differently; therefore, their perception should be measured using non-expert and simple-to-use methods [8,9]. One of the most widespread definitions of risk perception is contained in the standard ISO Guide 73:2009 Risk management—Vocabulary [10], which describes it as “stakeholder’s view on a risk”. Risk perception reflects the stakeholder’s needs, issues, knowledge, beliefs and values. This study adds to the knowledge of post-COVID-19 organizational risk level in Polish enterprises and quantitatively examines the scales of risk and risk management systems affecting operational risk and organizational performance. An exploratory research design is used to assess these scales. The scales are assessed using a pilot survey of Polish enterprises. A practical implication of this research is that the results may offer an insight into the managers’ perceptions of the risks associated with their new working environments and the roles of the presented categories of risk management. Using the survey results, risk management was generally perceived better than the level of operational risk, compared to the period before the pandemic. Therefore, the development and improvement of risk management allowed the researched organizations to survive and to deal with the pandemic crisis. They have been able to achieve their goals mainly by a slight reduction in risk appetite and lowering the tolerable risk threshold level. Even so, organizations have improved their ability to adapt and take advantage of opportunities.

## 2. Review of the Literature

**Figure 1:**
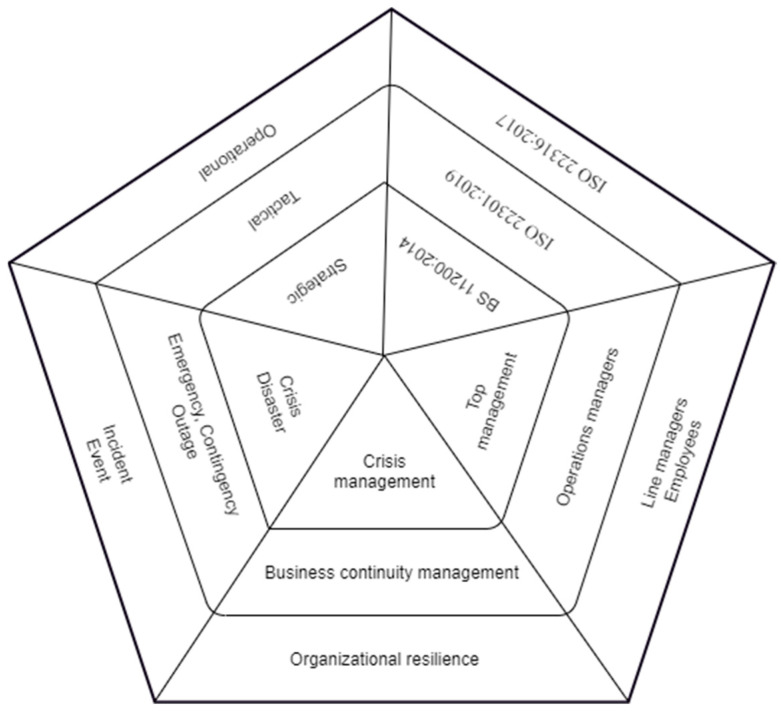
A standard-based concept of the integration of corporate recovery management systems based on a pentagonal pyramid. Source: [14].

An uncertain environment causes obstacles, such as incidents, disruptions, crises, or disasters, that can negatively affect an organization. The ability of an organization to resist any disruption requires three elements: organizational resilience (OR), crisis management (CM), and business continuity management (BCM). These elements create a coherent concept of the integration of corporate recovery management systems. Organizational resilience is the foundation of the whole concept, while crisis management is responsible for the coordination of all the concept components. Each element is established with its own standard. CM is based on BS 11200:2014 Crisis Management—Guidance and good practice [11], BCM is based on ISO 22301:2019 Business Continuity Management System Requirements [12], and OR is based on ISO 22316: 2017 Security and Resilience—Organizational Resilience—Principles and Attributes [13]. CM, BCM, and OR rely on and use risk assessment, and are based on the concept of continuous improvement, which allows the enhancement of the organization [14]. The integration of corporate recovery management systems is depicted on Figure 1.

**Figure 2:**
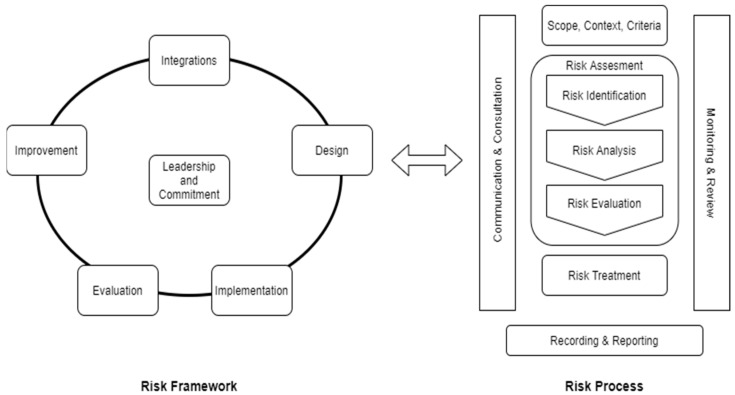
Risk management framework and process. An adaptation based on source [19].

To become more resilient, organizations should anticipate and respond to hazards and opportunities in both the internal and external environment. Occupational safety is an example of a risk in the internal environment. The better use of the hierarchy of control measures in an enterprise, the greater the company’s resilience. Thus, effective risk management builds organizational resilience in enterprises [13]. Good business practices also help to achieve it [15]. To manage the risk, there is a need to properly use necessary information on past and present disruptions and recognize the perception of the risk by the workers. This will allow for the improvement of the decision-making process correlated to the operational risk. The proactive processing of information needs modern technology. The concepts of the smart word and Industry 4.0 are very useful in this context. The smart world indicates modern technological solutions [16]. Industry 4.0 is perceived as an inventive strategy for production management which improves the efficiency and competitiveness of the organization [17]. Additionally, the concept of “sustainable development” is important to any enterprise that wants to grow [18]. Risk awareness and risk management are most useful to prevent and slow down the transmission of the COVID-19 pandemic and build organizational resilience. Management and other employees should be aware of the boundaries of acceptable and tolerable risk, and directly take preventive measures when this level is exceeded. There are many descriptions of risk. One of the most general in the economy is the definition contained in the standard ISO 31000: 2018 [19], which defines risk as “the impact of uncertainty on objectives”. In the same document, risk management is defined as “coordinated activities aimed at directing and controlling the organization in relation to risk”. The objective of risk management is the creation and protection of value. It enhances performance, fosters innovation, and helps the achievement of goals. According to ISO 31000: 2018, risk management is based on the principles of risk management, but above all on the risk management framework and the risk management process. The purpose of the risk management framework is to assist the organization in integrating risk management into significant activities and functions. Framework development encompasses integrating, designing, implementing, evaluating, and improving risk management across the organization. The risk management process involves the systemic application of policies, procedures, and practices to the activities of communicating and consulting, establishing context, and assessing, treating, monitoring, reviewing, recording, and reporting risk. The International Organization for Standardization (ISO) defines this standard as a framework for managing a variety of risks in various sectors of the economy. However, each of these sectors should adapt the relevant concepts to manage the specific risk. The risk management framework and process are depicted in Figure 2.

Complementing the ISO 31000 and the issues raised by this standard, is the ISO IEC 31010: 2019 Risk Management—Risk Assessment Techniques standard [20]. This standard provides a set of guidelines for the selection and the application of risk assessment techniques in a wide range of situations. These techniques are used as a decision aid in cases of uncertainty to provide information on individual risks and as part of the risk management process. On the other hand, the ISO Guide 73 [10] provides basic vocabulary to develop a common understanding of risk management concepts. It is worth emphasizing that these standards are universal and can also be used in occupational risk management in accordance with ISO 45001: 2018 Occupational Health and Safety Management Systems—Requirements and Guidelines for Use [21].

**Figure 3:**
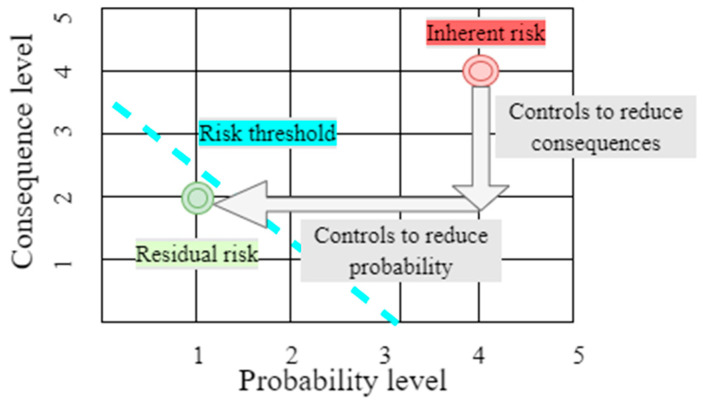
Inherent and residual risk idea in the risk reduction process. An adaptation based on source [15].

The literature on and approaches toward risk management distinguish between inherent risk and residual risk [22]. Inherent risk is the natural risk level without using controls to reduce consequences or probability. Even if all possible controls are implemented, residual risk remains after the management of the company takes action to minimize the impact and the probability of adverse events, including control actions taken in response to the risk [22]. In order to reduce the level of risk, the probability of risk occurrence and its consequences should be reduced or eliminated below the company’s risk threshold to an acceptably low level with as little residual risk as possible. This mechanism is presented in Figure 3. The general formula for calculating the residual risk can be presented as shown as in (1)

Residual Risk = Inherent Risk − Impact of Risk Controls(1)

where the overall concept of risk is consequence multiplied by probability [22].

One example of risk threshold definition is as follows: “quantified measures that represent upper and lower limits of acceptable tolerance around objectives” [23]. The threshold should be checked against risk capacity, which is defined as “Ability of an entity to bear risk, quantified against objectives” [23].

**Figure 4:**
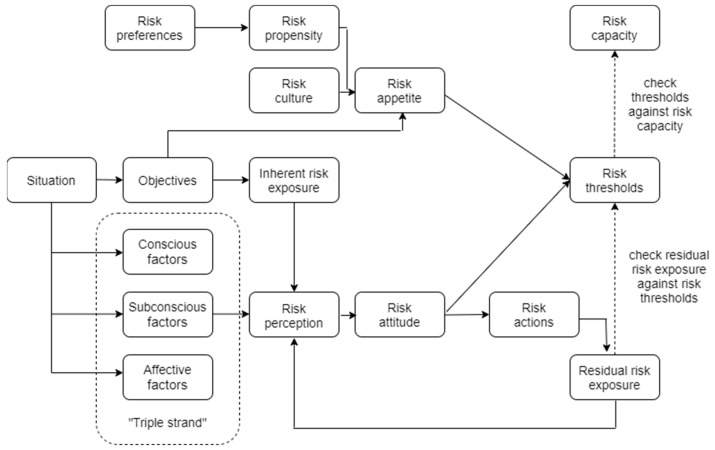
The Risk Appetite–Risk Attitude (RARA) model. An adaptation based on source [23].

Despite operational risks, organizations must take risks to achieve their objectives. An organization’s risk appetite is the willingness to accept the risk to achieve objectives or the utilization of opportunities. The degree of uncertainty that an organization or a person is willing to bear in exchange for benefit is called risk appetite. Risk appetite guides risk management and the criteria by which a company decides to take risk [24]. The nature, importance and appetite for risk vary across the life cycles of organizations and the life cycles of projects [18]. In risk management and decision-making organization, risk appetite is considered to be an important factor. The risk appetite enables decision makers at all levels of the organization, from top management to operations managers and line managers, to define the assumed level of risk in a given situation [23]. One consequence of the 2008 global recession is that corporate governance regulators now require companies across a range of industries to clearly describe their risk appetite [22]. Most of the existing research on risk appetite has been conducted in economics, finance, and hospitality management [23]. From a scientific point of view, it is interesting to explain human and business risky behavior and risk attitude. Executive risk management varies according to the monitoring mode and can exhibit both tendencies to seek risk and risk aversion [24]. One example of a risk attitude definition is as follows: “Chosen response of an individual or group to a given risky situation, influenced by risk perception” [23]. In turn, according to the same author, risk perception is defined as a “View of the risky situation by individual or group, influenced by ‘triple strand’ (conscious, subconscious and affective) factors”. Risk perception, revenue, and mood are important factors affecting individual risky behavior. In turn, the risk propensity of the top management and income affect a company’s risk-taking behavior. The history of an organization’s risk taking and risk culture determines the attitude towards risk, and ultimately has an impact on the organization’s risk appetite [25,26,27]. An example of the use of risk appetite and risk attitude to support appropriate risk taking is the so-called Risk Appetite–Risk Attitude (RARA) model developed by David Hillson and Ruth Murray-Webster [23] (characterized in Figure 4).

The RARA model shows how both risk appetite and risk attitude influence the setting of risk thresholds and how important risk perception is in the decision-making process. According to the authors of the RARA model, it is possible to use a managed approach to select the appropriate risk attitudes in order to optimize both the decision-making process and the decision consequence. Decision result is directly related to organizational performance. Organizational performance is the ability of an organization to reach its goals. Pierre et al. claimed that organizational performance includes three specific areas of firm outcomes: (a) financial performance (profits, return on assets, return on investment, etc.); (b) product market performance (sales, market share, etc.); and (c) shareholder return (total shareholder return, economic value added, etc.) [28]. Thus, it can be concluded that risk management (both its structure and process) greatly impacts organizational performance. The key factors for effective risk supervision are the proper determination of risk appetite, an effective communication structure, and an effective risk control structure.

Risk management may be applied at different levels, e.g., strategic or operational ones. Strategic risks are risks that affect or are formed by an organization’s business strategy and strategic objectives. They include geopolitics, competition, technology, and customers. Operational risks are risks that affect an organization’s ability to achieve its strategic plan. They include markets, people, finance, and projects. The occupational risk related to the potential losses of workers’ lives and health is included in the operational risk. An operational (loss) event is defined by Haubenstock as “an event due to failed or inadequate processes, people or systems, or exposure to external events that caused, or potentially could have caused, a material loss or adversely impacted stakeholders” [29]. Using this term, The Basel Committee on Banking Supervision (BCBS) defines the operational risk as the “risk of loss resulting from inadequate or failed internal processes, people and systems or from external events” [30]. This definition includes human error, fraud, system failures, problems related to personnel management, commercial disputes, occupational accidents, fires, floods, etc. Due to the wide range of hazards, the authors decided to use this taxonomy as a common approach to operational risk in business. The composition of the components of operational risk taxonomy is depicted in Table 1.

## 3. Materials and Methods

The knowledge of categories of operational risk (BCBS taxonomy) and categories of risk management (ISO 31000: 2018, the standard-based concept of the integration of corporate recovery management systems and the RARA model) were used to prepare a survey questionnaire. The questionnaire was prepared to identify the impact of a pandemic on the organizational risk perception in an organization and to examine the impact of broadly understood risk management on organizational performance in a post-COVID-19 work environment. The questionnaire contained 28 questions, each rated on a 7-point Likert scale (from “strongly disagree” to “strongly agree”). These questions were then selectively allocated to individual scales of organizational risk perception: 11 questions were based on risk categories (IEF, EPWS, CPBP, BDST, EDPM and Total OR—DPA was excluded to examine its impact on the total and rest organizational risk scales and on risk management perception) and 17 questions were based on organizational performance, risk management processes, risk management framework, recovery management systems, and Total Mgmt.

After developing a tool for assessing risk scales, there was an attempt made to initially validate the tool. The first step in validating the survey was to establish face validity. The questions were assessed by two university employees to evaluate whether the questions effectively captured the topic under investigation and to check the survey for common errors such as double-barreled and leading questions [31].

The second step was to pilot test the questionnaire on a subset of the intended population. The questionnaire was accompanied by a cover letter, explaining the purpose of the research. Participation in the study was voluntary and anonymous. The questionnaire included an information clause about the possibility of withdrawing from the study at any time, the motivation for the study, and where and how the results would be stored. Due to the type of survey relating to the evaluation of the organization and not personal characteristics, there were no risks associated with completing the questionnaire for the respondent, which was communicated to the study participants. Participants did not make any comments on the method and tools of the survey, as they were instructed in the introduction. In view of the above-described characteristics of the research and the provisions of the regulations on the work of the Commission on Ethics of Scientific Research conducted with the participation of people at Poznan University of Technology, research was not subject to opinion. Moreover, the research began before the commission was appointed by Rector’s Order RO/IV/15/2022. The questionnaire was aimed at managers of all three levels of companies (strategic, operational, and line). Due to the area of research, the specificity of enterprises and the current epidemic situation, which significantly hindered direct contact with the respondents, the authors decided to use both the PAPI (Paper and Pencil Interview) and CAWI (Computer-Assisted Web Interview) methods. The surveyed companies’ managers who had direct access to a computer with the Internet received a questionnaire prepared in Google Forms, and for the remaining managers, parcels were sent containing paper questionnaires. The authors transferred the completed paper questionnaires to the Google Form on their own. The data collection procedure was conducted in the period between 28 February 2022 and 5 April 2022. The study period in Poland coincided with the extinction of the fourth wave of COVID-19 cases, which has been perceived as the most severe so far.

In terms of evaluating the research sample conducted, it should be noted that there were 2,200,000 enterprises registered in Poland in 2021 [32]. The size of surveyed companies was assumed on the basis of [33], as follows: micro, 1–9 employees; small, 10–49 employees; medium, 50–250 employees; and large, over 250 employees. According to [32], 96.9% of enterprises are micro, where due to obvious reasons there is no clear division in the three levels of management structure. Thus, the authors decided to exclude them from the research. This means that the overall population was 68,200 enterprises. In terms of the size of the companies, there were 48,400 small companies (71%), 15,400 medium enterprises (23%), and 4400 large companies (6%). The percentage of employees who are managers in these companies was assumed on the basis of [34] at the level of approximately 13%. Moreover, the authors simplified the average managerial structure in the companies as follows:

- The median value of employees in a small company was assumed at 45, with the management structure: 1 top manager, 2 operational managers, and 4 line managers.
- The median value of employees in a medium company was assumed at 160, with the management structure: 3 top managers, 6 operational managers, and 12 line managers.
- The median value of employees in a large company was assumed at 350, with the management structure: 5 top managers, 10 operational managers, and 30 line managers.

Thanks to these assumptions, it was possible to assess the population of top managers, operational managers, and line managers in the presented companies. The results are as follows: the overall population of managers is 860,200, including 116,600 top managers (14%), 233,200 operational managers (27%), and 510,400 line managers (59%).

The criterion of level of management (top managers, operational managers, and line managers) provided rare subgroups for which a sampling frame does not exist. In this case, probability sampling would not work but quota sampling could achieve sufficient numbers to analyze and emphasize differences [35]. Thus, the authors decided to choose quota sampling as the type of non-probability sampling method. The aim of quota sampling is to control the composition of the final achieved sample ‘by design’. The design may duplicate the actual composition of the population of interest, have equal numbers of different types of respondents, or exceed the sample of a definite type of respondent [35].

The selection of the sample for the research consisted of four steps to form a quota sample.

(1) Division of the sample population into subgroups. The study included the following subgroups of respondents: the company size criterion: small, medium, and large; the managerial level criterion: top, operational, and line.
(2) Determining the weight of the subgroups. The authors evaluated the proportions of the subgroups as follows: the company size criterion: equal number of different types of respondents (due to the relatively weak management structure in small companies, it was decided to reduce their participation from 71% to 33% and strengthen the number of medium-sized companies from 23% to 33% and large companies from 6% to 33%); the managerial level criterion: replicate the actual composition of the population as follows: top (14%), operational (27%) and line (59%).
(3) Selection of an appropriate sample size. The sufficient sample of the survey was calculated according to the managerial level criterion using the sample size calculator [36]. The calculator utilizes the central limit theorem and the sample size calculation uses Formula (2):

(2) $$n=\frac{z^{2}\times \;\overset{⌢}{p}\;(1-\;\overset{⌢}{p}\;)}{\varepsilon^{2}}$$

where:

n is sample size;
z is the z score;
ε is the margin of error;
$\overset{⌢}{p}$ is the population proportion

Due to the assumption of conducting a pilot study, a 90% confidence level and a 90% confidence interval were established. As a result, a sufficient sample of 150 responders was calculated including top managers (32 responders), operational managers (53 responders), and line managers (65 responders), changing the pre-defined weight of the subgroup related to managerial level criterion (top from 14% to 21.33%, operational from 27% to 35.33%, and line from 59% to 43.33%, respectively).

(4) Conducting the survey in accordance with the defined quotas.

The defined quotas are presented in Table 2.

The selection of the sample was based on data obtained from the Internship and Career Centre of Poznan University of Technology regarding business entities registered for cooperation with the University as of 31 December 2021. The authors selected the companies that met the company size criterion from the list, and used them as the sampling frame. Respondents were selected from the sampling frame of companies according to a fixed periodic interval: a random start between 1 and 4 and every 4th respondent thereafter (2, 6, 10 …). The authors assumed that the study was a screening of the overall population of managers in Polish companies and, therefore, assumed one respondent for each company, taking into account a quota of the managerial level criterion. Finally, the questionnaire was sent to 150 companies in Poland. A total of 47 completed questionnaires were received during the research, which are characterized in Table 3. The average response rate was 31.33%.

After responders filled out the form, they tried to point out which questions were weak or irrelevant. Two questions were dropped due to low rate of item total correlations (a correlation between the question score and the overall assessment score).

The results for non-response in the context of the level of managers were not practically significantly different (top—68.75%, operational—56.60%, and line—78.46%, respectively). Many studies have shown, due to declining response rates, a weak relationship between response rates and non-response bias [35]; therefore, the authors considered the results sufficient in terms of quality. However, the survey has some limitations described in the discussion section.

The last step of the validation was checked in response to the internal consistency of questions belonging to the same scales. First, the normal distribution of the variables was examined by the Shapiro–Wilk test. The test achieved statistical significance (*p* < α), which confirms the distribution deviating from the Gaussian curve (the level of significance = 0.05). Thus, the Spearman correlation coefficients for internal consistency were used. The total item correlation oscillated from 0.11 to 0.63. The strength of the correlation ranged from negligible (0.0–0.10) to very strong (0.7–0.79), while the values were mostly in a moderate, strong, or very strong interval of strength [37]. As a tool to measure the questions’ reliability, the overall Cronbach’s alpha coefficient was employed. It reached the value of 0.83, confirming that the questionnaire is reliable for data evaluation [38]. The dataset was processed with the Statistica 13 application. The results of the semi-qualitative assessment were utilized in this questionnaire.

## 4. Results

The following questions represent the managerial perception of risk in an organization in a post-COVID-19 work environment. The item total correlation differed. The results of the internal consistency analysis are shown in Table 4.

For a better understanding of operational risk perception and risk management perception areas and their further analysis, the results are presented separately for operational risk perception in Table 5 and Table 6, and for risk management perception in Table 7 and Table 8, respectively. The obtained results were further elaborated in order to find mean values and item total correlations for calculating the IEF, EPWS, CPBP, BDST, EDPM and Total OPS scales according to BCBS taxonomy (Table 5).

Table 6 shows the calculated results of the Spearman correlation between the IEF, EPWS, CPBP, BDST, EDPM and Total OPS scales.

The obtained results were further elaborated in order to find mean values and item total correlations for calculating the Total OPS, Organizational performance, Risk Mgmt., process, Risk Mgmt. framework, Recovery Mgmt. systems, and Total Mgmt. scales. They are depicted in Table 7.

Table 8 shows the calculated results of the Spearman correlation between the Total OPS, Organizational performance, Risk Mgmt. process, Risk Mgmt. framework, Recovery Mgmt. systems, and Total Mgmt. scales.

## 5. Discussion

The presented research is the starting point for managing the organization’s operational risk. The goal of the survey was achieved. The main dependencies were established, both in terms of operational risk perception and risk management scales. When it comes to the perception of operational risk, the overall level of inherent risk (Total OPS) was rated slightly higher (3.69) by respondents compared to the period before the pandemic (see Table 5). On the basis of the survey, five scales related to IEF, EPWS, CPBP, BDST, and EDPM were prepared. Additionally, the Total OPS scale was proposed in order to compare aggregate results (mean of IEF, EPWS, CPBP, BDST, and EDPM). In this context, IEF was assessed as the best (4.03). It exceeded the other risk scales of EPDM (3.98), EPWS (3.72), CPBP (3.56), and BDST (3.37). Worse situations with higher risk were connected with losses arising from disruptions to or failures in systems, tele-communication and utilities. Understanding these failure modes allows for preparation efforts critical to organizational continuity [39]. These issues were often revealed by business continuity problems, which forced companies to develop recovery management systems (see Table 7). The average response results were calculated in the context of the size of employment (10–49, 50–250, and 250+), and the level of management (top, operational, and line). The best results in terms of the lower level of risk were related to the size of the company (small organizations (3.76), large organizations (3.69), and medium ones (3.68)). It can be concluded that, actually, there is a slight difference in the operational risk perception level in terms of size of employment. Moderately poorer risk perception scores compared to the pre-pandemic period created a balance between the risks and opportunities perceived by management. This is confirmed by the results published by PARP (The Polish Agency for Enterprise Development) on the condition of small- and medium-sized enterprises in Poland: both the level of newly established companies and the number of liquidated ones indicate slight upward trends [40].

In terms of the level of management, the level of risk was better assessed by line managers (3.90), operations managers (3.74), and top managers (3.29). The worse results for top managers were related to more critical approaches due to more holistic knowledge of the internal and external risks of organizations in the post-COVID-19 work environment. This knowledge comes from the fact that top management is obliged to look at risks from a strategic horizon point of view [39]. It is worth adding that the occupational risk level was assessed slightly higher by respondents compared to the period before the pandemic (3.87) (see Table 5). Still, the existing pandemic and, therefore, the possibility of adverse impacts on the health of employees exposed to occupational health hazards, explain the prudent approaches of companies toward this issue [9]. Situations with employee relations (3.66), and discrimination (3.64), i.e., in the context of vaccinations, also became worse. In addition, the Spearman rank order correlations between IEF, EPWS, CPBP, BDST, EDPM, and Total OPS were calculated (see Table 5). On the basis of the calculations, strong and very strong correlations between the parameters were found. The results of the pairs were as follows: EPWS/IEF—0.69, EPWS/CPBP—0.54, EPWS/BDST—0.53, EPWS/EDPM—0.53, and EPWS/Total OPS—0.86. This means that the scales are strongly correlated and complement each other, creating a coherent concept of BCBS taxonomy. It can be concluded that the highest correlation was the pair of EPWS/Total OPS. This shows the important role of EPWS risk in Total OPS risk score.

When it comes to the perception of risk management and operational performance, in contrast to the perception of operational risk, the overall level of assessment was slightly better (4.77) (Total in Table 7) compared to the period before the pandemic. On the basis of the survey, four scales related to the organizational performance, risk management process, risk management framework, and recovery management systems were prepared. Additionally, the Total Mgmt. scale was proposed in order to compare the aggregate results (mean of the organizational performance, risk management process, risk management framework, and recovery management systems). In this context, the Risk Mgmt. framework was assessed as the best (5.00). It exceeded the other scales of Recovery Mgmt. systems (4.77), organizational performance (4.66), and risk management processes (4.61). These moderately optimistic results show that the surveyed organizations most likely have a risk management policy or specific rules. They have been following these rules for some time, thanks to which they achieve relatively positive organizational performance results. The surveyed organizations also had information relevant to improvements in risk management. They implemented elements of the risk framework and risk management process to integrate risk management into the governance of the organization, including decision-making, thanks to which the enterprises achieved their objectives [19]. The risk attitude was influenced by operational risk perception and adequate risk treatment (see Figure 4). Due to higher levels of operational risk perception score compared to the period before the pandemic (3.69), the most common risk treatment method (see Table 4) was assessed as risk reduction (5.11). It exceeded the other methods of risk sharing (3.91) and risk avoiding (3.87). Organizational performance achieved a relatively good score by a slight reduction in risk appetite and a lower tolerable risk level threshold (4.66). Even so, organizations have improved their ability to adapt and take advantage of opportunities (5.04). It is also worth emphasizing that the enhancement and improvement of recovery management systems allowed them to deal with the COVID-19 pandemic crisis situation [14]. Thus, the greatest improvement in relation to the state before COVID-19 was recorded in crisis management knowledge and skills (5.06). This exceeded the other management aspects of risk management (4.89), business continuity management (4.64), and organizational resilience (4.49). The average response results were also calculated in the context of company size (10–49, 50–250, and 250+) and managerial level (top, operational, and line). The best, in terms of the perception of risk management, was large organizations (4.90), followed by small organizations (4.85) and medium ones (4.63). In terms of risk management level, it was better assessed by top managers (5.03), then line managers (4.82) and ops managers (4.63). It can be concluded that, actually, there is a slight difference in risk management perception in terms of the risk management level. In addition, the Spearman rank order correlations between organizational performance, risk management process, risk management framework, recovery management systems, and total were calculated (see Table 8). On the basis of the calculations, moderate, strong and very strong correlations between the parameters were found. The results of the pairs were as follows: organizational performance/risk management process—0.43, organizational performance/risk management framework—0.58, organizational performance/recovery management systems—0.73. This means that the scales are strongly correlated and complement each other, creating a coherent concept of risk management. It can be concluded that the highest correlation was the pair of risk management framework/Total Mgmt. (0.87). This shows the important role of the risk management framework in total risk management score.

This research comes with some limitations. Due to the choice of a non-probability sampling method, the authors selected samples based on their subjective judgment rather than random selection (however, the draw of companies was made). The research was exploratory. The authors used controlled quota sampling. This imposed restrictions on the authors’ choice of samples. Additionally, in the survey, the authors simplified the median value of managerial structure, and the managers of organizations were asked to assess the management issues. It may be considered that there could have been a selection bias, but the authors recognized them as risk experts who had sufficient experience and knowledge about risk assessment processes. It was mentioned in the Materials and Methods section that the companies’ data were obtained from the Internship and Career Centre of Poznan University of Technology. This may also be considered as a selection bias, but to compensate for it, the authors used hybrid approaches: probability-based selection and quota sampling. The authors assumed that the study was a screening of the overall population of managers in Polish companies, and therefore assumed one respondent for each company (taking into account a quota of the managerial level criterion). We understand that there would be added value in looking at three different managerial levels inside each company separately. Due to these limitations, additional surveys should be taken. As a future study, each company and its structure can be considered an individual source of data during quality assessment, using in-depth interviews of three levels of managers.

Furthermore, the average response rate was relatively low, but due to a weak relationship between response rates and non-response bias, we considered the results sufficient in terms of quality. The quota sampling aimed to obtain the best representation of respondents in the final sample. Thus, the authors are convinced that the surveyed companies in the pilot study provide sufficient statistical representativeness for a preliminary discussion on the managerial perception of risk in organizations in a post-COVID-19 work environment.

## 6. Conclusions

The purpose of the study was to examine the impact of a pandemic on organizational risk (including occupational risk) perception in an organization by managers of all three levels (strategic, operational, and line) and to examine the impact of broadly understood risk management on organizational performance in a post-COVID-19 work environment. Using the survey results, some scales, compared to the period before the pandemic, e.g., Total OPS, EPWS, CPBP, BDST, and EDPM, were perceived as worse, and some scales, e.g., IEF, Organizational performance, Risk Mgmt. process, Risk Mgmt. framework, Recovery Mgmt. systems, and Total Mgmt., were perceived as a better. Due to the fact that risk management was generally perceived as better than the level of operational risk, compared to the period before the pandemic, surveyed organizations have coped with the crisis. They could achieve their goals mainly by a slight reduction in risk appetite and lowering the tolerable risk level threshold. Even so, organizations improved their ability to adapt and take advantage of opportunities. It is also worth emphasizing that the improvement in recovery management systems strongly supports the process of dealing with the COVID-19 pandemic crisis situation. Thus, the greatest improvement in relation to the state before COVID-19 was recorded in the crisis management skills of organizations. Based on the calculations, a strong correlation was found between the operational risk perception scales and the risk management perception scales, respectively. This means that the scales are strongly correlated and complement each other, creating a coherent concept. It can be concluded that the highest correlation was the pair of EPWS/Total OPS (0.86). This shows the influence of occupational risk on Total OPS risk score. For companies, it is crucial to ensure their ability to protect workers’ lives and health in a crisis. As far as correlation in risk management perception is concerned, the highest correlation was the pair of risk management framework/Total Mgmt. (0.87). This shows the influence that risk management framework has on total risk management score.

As far as the perspective of the risk perception of three manager levels is concerned, we can state that it was the most important criterion of the study. We surveyed three levels of management in the organizational hierarchy to compare their perspectives on risk perception as well as risk management perception.

Top management set broad strategic goals for the organization and focused on the big picture. They know the external and internal context of the organization very well. They also are ultimately responsible for the organization’s performance. Thus, we can explain their perception of risk as the most critical among all three levels of managers (3.29). They assessed CPBP as the worst risk (3.13). This is probably due to the fact that CPBP is connected to strategic risks that include competition and customers. After all, top management has an obligation to look at risks from a strategic perspective [39]. At the same time, their perception of risk management in their companies was the most positive (5.03), followed by organizational performance level (5.10). The best score was for Risk Mgmt. framework (5.38). This is probably due to the fact that top managers are ultimately responsible for the overall level of risk management in the company and its performance, so they subjectively assess it best, although they are aware of a level of operational risk higher than before the pandemic. This does not change the fact that risk management and organizational performance are generally perceived quite well.

Operational managers are responsible for carrying out the goals set by top management. They do so by setting goals for their units. They also control, motivate and assist line managers in achieving business objectives. We can explain their perception of risk as moderate among all three levels of managers (3.74). They assessed BDST as the worst risk (3.54). This comes from the fact that they are usually responsible for business continuity, which has been a big problem during the pandemic. At the same time, their perception of risk management in their companies is also positive (4.63), as is their perception of organizational performance level (4.39). The best score was also for Risk Mgmt. framework (4.81). This proves the good integration of risk management principles in companies and good leadership [19].

Line managers are responsible for the daily management of workers who produce or provide services. They are largely managed by operational management. We can explain their perception of risk as the best among all three levels of managers (3.94). This is related to their high assessment of risk management in the company. They are convinced that any exposure to risk is suitably understood and managed [41]. They assessed BDST as the worst risk (3.14). This comes from the fact that they probably often participated in business continuity problems during the pandemic. At the same time, their perception of risk management in their companies is also positive (4.82), as is their perception of organizational performance level (4.79). The best score was also for Risk Mgmt. framework (5.03). This is additional evidence of the good practice of organizations in integrating risk management into significant activities and functions. Effective risk management policies and practices increase stakeholder confidence, competitive advantage, and ultimately an organization’s long-term viability [42].

Generally, we observed similar tendencies of risk and risk management perceptions across all three manager levels in the post-COVID-19 work environment. The values of the results also did not differ significantly. In other words, a holistic management perspective across all three levels of management has been adopted in the surveyed companies to ensure success [42]. Today, the trend is towards a coordinated, interactive, company-wide approach that assesses and manages all risks together [43,44]. This holistic approach enhances silo-based traditional risk management [42]. Risks are related in the present day, and need to be understood in context. During the COVID-19 pandemic, the risk started with DPA and then cascaded with other operational risks, affecting EPWS, BDST, CPBP, and other risks. The key was awareness of the new challenges resulting from the pandemic by top management, as well as good communication and cooperation among the three management levels. This proves that, during the pandemic, the organizations improved risk management systems in terms of processes and structures. They also substantially improved the operation of recovery management systems, in particular crisis management. Thanks to this, the companies were able to enhance their organizational resilience, maintain organizational continuity, and ensure the company’s ability to protect workers’ lives and health in a crisis, and even slightly enhance organizational performance.

A practical implication of this research is that the results may offer an insight into the managers’ perceptions of risks associated with their new working environment and the roles of the presented scales of risk and risk management. Risk awareness and risk management are critical success factors for companies to survive a crisis or disruption. Undoubtedly, the COVID-19 pandemic was an opportunity to enhance these issues within organizations. Managers took into account the lessons learned from the crisis and use them in the continuous improvement process. This helps to enhance risk management and to develop an effective risk assessment for better decision-making, and hence improve the operational performance of the organization during disruptions. This provides a premise for coping with disruption at all three levels of company management in a post-COVID-19 work environment.

## Figures and Tables

**Table 1:** The BCBS taxonomy of operational risks.

Risk Categories	BCBS Definition	Sub-Categories/Examples
Internal and external fraud (IEF)	Losses due to acts of fraud involving at least one internal or external party	Account take-over and impersonation • Bribes and kickbacks • Forgery • Credit fraud • Insider trading (not on firm’s account) • Malicious destruction and misappropriation of assets • Tax noncompliance • Theft • Extortion • Embezzlement • Robbery • Intentional mismarking of position • Unauthorized and unreported transactions, computer hacking • Theft of information • Forgery • Theft
Employment practices and workplace safety (EPWS)	Losses arising from violations of employment and health and safety laws.	Discrimination • Compensation and termination issues • Health and safety issues and general liability
Clients, products and business practices (CPBP)	Losses arising from failure to meet obligations to clients or from the design of a product	Disputes over advisory services • Violation of anti-monopoly rules and regulations • Improper trade • Insider trading on firm’s account and market manipulation • Money laundering • Unlicensed activity • Product defects • Exceeding client exposure limits • Account churning aggressive sales • Breach of privacy • Misuse of confidential information • Customer discloser violation
Damage to physical assets (DPA)	Losses arising from damage inflicted on physical assets by a natural disaster or another event	Terrorism • Vandalism • Natural disasters (e.g., the COVID-19 pandemic)
Business disruption and system failures (BDST)	Losses arising from disruptions to or failures in systems, telecommunication and utilities	Hardware • Software • Telecommunications • Utility outage • Utility disruption
Execution, delivery and process management (EDPM)	Losses arising from failed transaction processing with counter-parties such as vendors	Incorrect client records • Negligent loss or damage of client assets • Unapproved access to accounts • Client permissions • Missing and incomplete legal documents • Failed mandatory reporting obligations • Inaccurate external reports and non-client counterparty disputes • Accounting errors • Collateral management failure • Data entry, maintenance or loading error • Delivery failure • Miscommunication • Missed deadlines • Vendor dispute

An adaptation based on source: [30]

**Table 2:** Features of the defined quotas.

Size of Company	Top Mgmt.	Ops Mgmt.	Line Mgmt.	All
Small	12	21	17	50
Medium	10	17	23	50
Big	10	15	25	50
All	32	53	65	150

**Table 3:** Features of the research subjects with response rates.

Size of Company	Top Mgmt.	Ops Mgmt.	Line Mgmt.	All
Small	3 (25.00%)	7 (33.33%)	2 (11.76%)	12 (24.00%)
Medium	1 (10.00%)	7 (41.18%)	5 (21.74%)	13 (26.00%)
Big	6 (60.00%)	9 (60.00%)	7 (28.00%)	22 (44.00%)
All	10 (31.25%)	23 (43.40%)	14 (21.54%)	47 (31.33%)

**Table 4:** Results of the internal consistency analysis.

No.	Rated Item	Mean	SD	Item Total Correlations
1.	In our organization, the level of risk related to internal theft and fraud has significantly decreased (compared to the period before the pandemic).	4.06	1.57	0.39
2.	In our organization, the level of risk related to external theft and fraud has significantly decreased (compared to the period before the pandemic).	4.00	1.60	0.52
3.	In our organization, the level of risk related to employee relations has significantly decreased, e.g., compensation payments, strikes, and disclosure of employees’ personal data (compared to the period before the pandemic).	3.66	1.51	0.47
4.	In our organization, the level of risk related to employee safety in the work environment has significantly decreased (compared to the period before the pandemic).	3.87	1.74	0.26
5.	In our organization, the level of risk related to division and discrimination has significantly decreased (compared to the period before the pandemic).	3.64	1.63	0.43
6.	In our organization, the level of risk related to customer service has significantly decreased, e.g., breach of customer trust (compared to the period before the pandemic).	3.89	1.68	0.63
7.	In our organization, the level of risk related to inappropriate market practices, e.g., market monopolization, has significantly decreased (compared to the period before the pandemic).	3.55	1.32	0.51
8.	In our organization, the level of risk related to the quality of products or services provided has significantly decreased (compared to the period before the pandemic).	3.23	1.45	0.13
9.	In our organization, the level of risk related to business continuity disruption has significantly decreased (compared to the period before the pandemic).	3.34	1.77	0.21
10.	In our organization, the level of risk related to system errors, e.g., discontinuities in infrastructure operation, has significantly decreased (compared to the period before the pandemic).	3.40	1.56	0.47
11.	In our organization, the level of risk related to the transactions has significantly decreased (compared to the period before the pandemic).	3.98	1.48	0.27
12.	Our most common form of dealing with risk is sharing the risk with other entities, e.g., with insurers.	3.91	1.23	0.11
13.	Our most common form of dealing with risk is reducing it.	5.11	1.36	0.31
14.	Our most common form of dealing with risk is avoiding it, e.g., giving up a planned task related to that risk.	3.87	1.70	0.14
15.	In our organization, top management personally demonstrates their commitment to the risk management process.	4.87	1.31	0.30
16.	In our organization, the scope of the risk management process is defined comprehensively.	4.94	1.47	0.38
17.	In our organization, we have evidence for periodic reviews and monitoring of the results of the risk management process.	4.94	1.33	0.41
18.	Our organization has established clearly defined criteria for risk assessment (evaluating the probability of an event and its consequences) in the risk management process.	5.23	1.59	0.47
19.	Our organization has integrated risk management with the organizational structure of the organization.	4.98	1.42	0.44
20.	Our organization has adopted an approved communication and consultation approach to support the organization’s risk management process.	4.94	1.37	0.13
21.	Our top management has established a policy that clearly states the organization’s goals and its own commitment to the organization’s risk management process.	5.26	1.31	0.36
22.	In our organization, the appetite for risk and the associated tolerable risk level threshold (compared to the period before the pandemic) have significantly decreased.	4.66	1.59	0.46
23.	Our ability to manage risk has significantly increased (compared to the period before the pandemic).	4.89	1.31	0.49
24.	Our performance and operational results have significantly increased (compared to the period before the pandemic).	4.28	1.61	0.42
25.	Our ability to manage the business continuity of the organization has significantly increased (compared to the period before the pandemic).	4.64	1.39	0.41
26.	Our organizational resilience has increased significantly (compared to the period before the pandemic).	4.49	1.41	0.41
27.	Our crisis management skills have significantly increased (compared to the pre-pandemic period).	5.06	1.21	0.40
28.	Our ability to adapt and take advantage of opportunities has significantly increased (compared to the period before the pandemic).	5.04	1.23	0.51

**Table 5:** Scales of operational risk perception.

Scale	Item’s List	Top Mgmt.	Ops Mgmt.	Line Mgmt.	10–49	50–250	250+	Total	α CR	It-Tot Cor.
IEF	1–2	3.60	3.96	4.46	4.16	4.15	3.98	4.03	0.89	0.39–0.52
EPWS	3–5	3.27	3.81	3.90	3.72	3.69	3.79	3.72	0.85	0.26–0.47
CPBP	6–8	3.13	3.49	3.98	3.50	3.69	3.56	3.56	0.86	0.13–0.63
BDST	9–10	3.30	3.54	3.14	3.67	3.08	3.39	3.37	0.87	0.21–0.47
EDPM	11	3.20	4.26	4.07	4.00	3.85	3.86	3.98	0.88	0.27
Total OPS	1–11	3.29	3.74	3.90	3.76	3.68	3.69	3.69	0.83	0.13–0.63

**Table 6:** The results of the Spearman correlation between scales of operational risk perception.

Variable	IEF	EPWS	CPBP	BDST	EDPM	Total OPS
IEF	1.00	-	-	-	-	-
EPWS	0.69	1.00	-	-	-	-
CPBP	0.49	0.54	1.00	-	-	-
BDST	0.25	0.53	0.65	1.00	-	-
EDPM	0.37	0.53	0.43	0.57	1.00	-
Total OPS	0.73	0.86	0.83	0.76	0.67	1.00

**Table 7:** Scales of risk management and organizational performance perception.

Scale	Item’s List	Top Mgmt.	Ops Mgmt.	Line Mgmt.	10–49	50–250	250+	Total	α CR	It-Tot Cor.
Organizational performance	22, 24, 28	5.10	4.39	4.79	4.63	4.51	4.83	4.66	0.87	0.42–0.51
Risk Mgmt. process	12–14, 16, 18	4.78	4.6	4.51	4.66	4.48	4.78	4.61	0.90	0.11–0.47
Risk Mgmt. framework	15, 17, 19–21	5.38	4.81	5.03	5.19	4.80	5.08	5.00	0.88	0.13–0.43
Recovery Mgmt. systems	23, 25–27	4.85	4.61	4.98	4.82	4.69	4.86	4.77	0.90	0.40–0.47
Total Mgmt.	12–28	5.03	4.63	4.82	4.85	4.63	4.90	4.77	0.90	0.11–0.51

**Table 8:** The results of the Spearman correlation between scales of risk management and organizational performance perception.

Variable	Organizational Performance	Risk Management Process	Risk Management Framework	Recovery Management Systems	Total Mgmt.
Organizational performance	1.00	-	-	-	-
Risk management process	0.43	1.00	-	-	-
Risk management framework	0.58	0.68	1.00	-	-
Recovery management systems	0.73	0.27	0.46	1.00	-
Total Mgmt.	0.84	0.73	0.87	0.72	1.00

## Data Availability

The data presented in this study are available on request from the corresponding author.
